# Prioritising target behaviours for research in diabetes: Using the nominal group technique to achieve consensus from key stakeholders

**DOI:** 10.1186/s40900-016-0028-9

**Published:** 2016-04-06

**Authors:** Jennifer Mc Sharry, Milou Fredrix, Lisa Hynes, Molly Byrne

**Affiliations:** Health Behaviour Change Research Group, School of Psychology, NUI Galway, University Road, Galway, Ireland

**Keywords:** Diabetes, Research prioritisation, Public and patient involvement, Research engagement, Behaviour change, Intervention development

## Abstract

**Plain english summary:**

The behaviour of people with diabetes (e.g. taking medication) and the behaviour of doctors and other healthcare professionals (e.g. checking patients’ blood sugar) are important. Our research group wanted to select one patient behaviour and one healthcare professional behaviour as topics to research in Ireland. Patients and healthcare professionals are not usually asked to help decide on research topics. In this study, we wanted to bring together patients, healthcare professionals and policy makers to help us decide on the most important target behaviours for research in diabetes in Ireland.

We worked with 24 participants, including people with diabetes, diabetes healthcare professionals and policy makers. First, participants suggested behaviours they thought were important to target for research in diabetes. Participants then attended a meeting and ranked which of the behaviours were the most important and discussed the results of the rankings as a group. We identified the most highly ranked patient and healthcare professional behaviours. The top ranked behaviour for people with Type 1 diabetes was to ‘take insulin as required’ and for people with Type 2 diabetes was to ‘attend and engage with structured education programmes’. ‘Engage in collaborative goal setting with patients’ was the top ranked behaviour for healthcare professionals.

Our study shows it is possible for researchers to work with people with diabetes, healthcare professionals and policy makers to decide on research topics. The top ranked behaviours will now be researched by our group in Ireland.

**Abstract:**

**Background**

Working with patients, healthcare providers, and policy makers to prioritise research topics may enhance the relevance of research and increase the likelihood of translating research findings into practice. The aim of the present study was to work with key stakeholders to identify, and achieve consensus on, the most important target behaviours for research in diabetes in Ireland.

**Methods**

Twenty-four participants, including people with diabetes, diabetes healthcare professionals and policy makers, took part in a nominal group technique consensus process. Through an online survey, participants generated lists of important target behaviours in three areas: managing Type 1 diabetes, managing Type 2 diabetes and preventing Type 2 diabetes. Participants then attended a research prioritisation meeting and ranked target behaviours in two rounds, with group discussion between ranking rounds. For each of the three key areas, the six top ranked behaviours relevant to people with diabetes and healthcare professionals were identified.

**Results**

In most cases, the most highly ranked behaviour was the same for Ranking 1 and Ranking 2 and consensus increased in relation to endorsement of top ranked behaviours. However, some behaviours did change position between rankings. The top behaviour relevant to people with Type 1 diabetes was ‘taking insulin as required’ and for people with Type 2 diabetes was ‘attending and engaging with structured education programmes’. ‘Engage in collaborative goal setting with patients’ was the top ranked behaviour relevant to healthcare professionals for managing both Type 1 and Type 2 diabetes. For preventing Type 2 diabetes, 'engage in healthy behaviours as a family' was the highest ranked population behaviour and ‘attend and engage with behaviour change training’ was the highest ranked professional behaviour.

**Conclusions**

It is possible to work with a diverse group of stakeholders to inform the diabetes research agenda. The priorities identified were co-produced by key stakeholders, including patients, healthcare professionals and policy makers, and will inform the development of a programme of behavioural research in diabetes in Ireland. The study also provides a worked example of a research prioritisation process using the nominal group technique, and identified limitations, which may be useful for other researchers.

## Background

There is strong evidence that changing people’s health-related behaviour can impact the leading causes of mortality and morbidity [1]. Behaviour change is central in the treatment of chronic illness, and targeting behaviours to prevent and manage chronic illnesses is imperative to deal effectively with increasing numbers of patients and escalating costs [2]. Recent examples of successful interventions have targeted both the behaviour of people with diabetes (diet and activity behaviours) and healthcare professional behaviour (early intervention for diabetes foot ulcers) [3, 4]. Changing behaviour can improve outcomes, with increasing evidence that interventions targeting behaviour change in diabetes can be effective [5, 6].

Despite the potential for behaviour change to improve diabetes outcomes, developing effective interventions is challenging. Diabetes management is complex, encompassing many different behaviours, and patients often struggle to make and maintain the behavioural changes required to manage their condition [7]. As this programme of behavioural research within diabetes continues to grow, how should we decide which behaviours should be prioritised for research? It has been suggested that much health-related research does not address topics which are of importance to patients and clinicians [8]. Seeking the views of patients and healthcare providers should be an essential part of determining the behavioural research agenda, especially to ensure impact from publicly-funded research [9].

Changing diabetes care to implement evidence from research into routine practice is a major challenge within the constraints of the healthcare system [10–12]. Within diabetes, attempts have been made to reduce the research-evidence gap with initiatives such as the Bringing Research in Diabetes to Global Environments and Systems (BRIDGES) project supporting the development of interventions that can be adopted and disseminated in real world settings [13]. Qualitative work with BRIDGES project researchers pinpointed lack of stakeholder and diabetes community links as key barriers in the implementation of diabetes research [14]. Despite the identified need to increase stakeholder engagement, there are few published examples of methods to involve stakeholders in the research process in diabetes.

More recently, efforts have been made to involve stakeholders in the research process by seeking input from patients and healthcare professionals in the prioritisation of research. One approach to collaborative research prioritisation has been driven by the James Lind Alliance which provides guidance on the development of Priority Setting Partnerships [15]. The James Lind Alliance Priority Setting Partnerships aim to bring patient and clinicians together to prioritise treatment uncertainties for research. Treatment uncertainties have been identified and ranked for a range of conditions [16] including Type 1 diabetes [17].

The first step in James Lind Alliance Priority Setting Partnerships is to identify potential research questions of interest to patients and providers. However, involving service users solely in the prioritisation of research questions can be limiting, with evidence that patient suggestions frequently fail to meet the criteria of a researchable question [18]. In addition, focusing solely on treatment uncertainties can limit the scope of research prioritisation. Research exploring the translation of evidence-based behaviour change interventions into practice, for example, does not fall within the treatment uncertainty remit.

The aim of our research prioritisation was to move beyond a more narrow discussion of treatment uncertainties to identify and achieve consensus on shared priority areas for research in diabetes in Ireland. Both the Delphi and nominal group technique processes have been used for the development of consensus in health services research. We also wanted to increase engagement in our programme of research and to build links with relevant stakeholders in Ireland, and so the anonymous approach associated with the Delphi technique was not appropriate to our aims.

The nominal group technique is a controlled group process for the generation and ranking of ideas and for consensus development [19]. The nominal group technique generates a high number of ideas and includes social interaction and discussion while limiting normative pressure for conformity through the use of private individual ranking [19]. Each participant in a nominal group technique process has an equal private ranking vote which provides a democratic process to navigate mismatches between researcher, patient, provider and policy maker priorities [15]. The nominal group technique also avoids limiting patients’ contribution to priority setting by not requiring priorities to be articulated in the form of a researchable question [18].

The aim of the present study was to engage in a nominal group technique processwith key stakeholders to identify, and achieve consensus on, the most important target behaviours for research in diabetes in Ireland. By identifying behaviour change targets that address the needs of the diabetes community, we hoped to enhance the relevance of our programme of research to the Irish healthcare context and to increase the likelihood of future translation of the findings into practice. By focusing on target behaviours rather than tightly defined research questions or treatment uncertainties, we hoped to maximise the potential for patients and healthcare professionals to impact on the development of the research agenda. Finally, by clearly outlining a systematic approach to engaging with key stakeholders we hoped to provide a useful resource for other researchers seeking to engage patients, professionals and policy makers, in the design of research.

The study forms part of a programme of research to develop and evaluate two behaviour change interventions in diabetes in Ireland: one focusing on a behaviour relevant to people with diabetes, and one on a behaviour relevant to healthcare professionals. In identifying high priority behaviours for research, we focused on three key areas: managing Type 1 diabetes mellitus (Type 1 DM), managing Type 2 diabetes mellitus (Type 2 DM) and preventing Type 2 DM. Within each of these areas, we aimed to develop a prioritised list of the most highly ranked target behaviours relevant to people with diabetes and healthcare professionals. We aimed to get the views of people with diabetes, healthcare professionals with clinical experience of diabetes and policy makers working in the area of diabetes.

## Methods

The nominal group technique (also known as an expert panel) was used to identify, and achieve consensus on, the most important target behaviours for research [20]. The nominal group technique was chosen as a systematic process that facilitates both idea generation and consensus development [19].

### Participants

The nominal group technique is as a small group technique, recommended for use in groups of up to ten participants [19]. Our sample size was informed by a desire to maintain the group dynamic of the technique while still including a range of healthcare professional, patient and policy stakeholders. We set a limit of 25 total participants, to allow for manageable group feedback and discussion as part of the nominal group technique process. Participants were sampled purposively to represent the following groups: people with Type 1 DM and Type 2 DM, healthcare professionals with clinical experience of diabetes and policy makers working in the area of diabetes. Potential healthcare professional and policy maker participants were identified through peer consultation. Those who declined were asked to nominate an alternative in their place. People with diabetes were informed of the study through a flyer circulated through Diabetes Ireland, a national charity dedicated to supporting people with diabetes. Details of number of patients contacted were not available to the research team. Response was generally enthusiastic, ten patients, and an additional two parents of children with diabetes, contacted the research team. Parents of children with diabetes were not eligible for participation as the focus of this prioritisation exercise was on adult patients with diabetes. The research team sent on full details of the meeting, including the proposed date, to potential participants and answered any questions. Six of the ten participants were available and attended the meeting. As participants were engaged in public and patient involvement activities and contributing to research design rather than taking part in a research study, ethical approval was not sought.

### Procedure

**Fig. 1:**
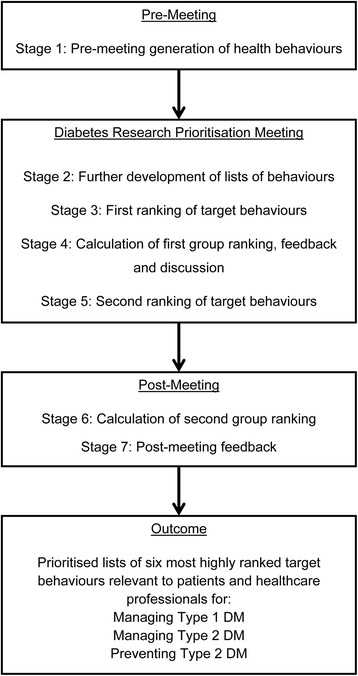
Diabetes Research Prioritisation Flow Chart

The nominal group technique begins with eliciting participant views on a topic. Similar suggestions are grouped together and a facilitated group discussion during a structured meeting allows for the clarification and evaluation of items. Each participant then privately ranks each item, the overall rankings are calculated, presented, and discussed and the items are privately re-ranked. The process of achieving consensus through an initial ranking, group discussion, and a second re-ranking was decided in advance and follows published nominal group technique guidance [19]. For the current study, a diabetes research prioritisation (DRP) meeting was organised in Galway, Ireland in October 2014. The study process is shown in Fig. 1 and each of the nominal group stages is outlined further below.

#### Pre-meeting

##### Stage 1: Pre-meeting generation and collation of health professional and patient behaviours

In advance of the DRP meeting, participants completed an online survey to generate lists of behaviours to target for research. Participants were asked to generate three health professional behaviours and three patient behaviours in each of the key diabetes areas (managing Type 1 DM, managing Type 2 DM, and preventing Type 2 DM). The importance of participants’ own views was emphasised and specific examples were provided for each category of behaviours.

The survey was administered using the Survey Monkey online tool and sent to participants one month in advance of the DRP meeting; a reminder was sent to all participants a week before the meeting. The research team collated all submitted responses by combining similar behaviours to avoid duplication, creating unique behaviours where original submissions included multiple behaviours and creating total lists of behaviours for each key diabetes area.

#### DRP meeting

##### Stage 2: Further development of lists of health professional and patient behaviours

**Fig. 2:**
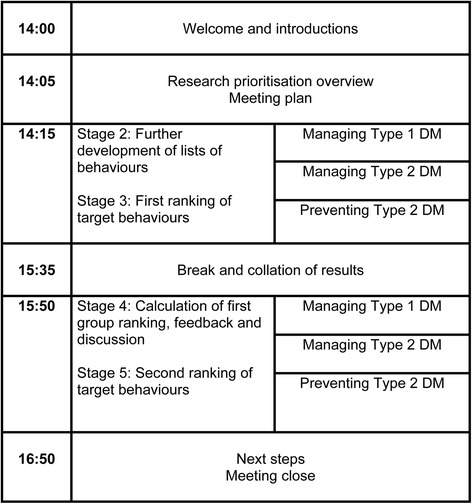
Diabetes Research Prioritisation Meeting Outline and Timings

Participants attended in person at a three hour DRP meeting and joined small group tables of four at random as they arrived. At the start of the meeting, the research team gave a short presentation outlining the format of the meeting and defined key terms. Participants were provided with the pre-generated lists of target behaviours and engaged in small group discussions to identify any additional behaviours. Small group tables included a mix of patients, healthcare professionals and policy makers. Each small group had an opportunity to feedback additional behaviours to the larger group, and these were added to the total lists. Group discussions were chaired by an experienced facilitator who aimed to ensure everyone had an opportunity to speak. This process was done six times during the meeting, for each of the three key diabetes areas (managing Type 1 DM, managing Type 2 DM, and preventing Type 2 DM), first for patient behaviours and then for health professional behaviours (see Fig. 2).

##### Stage 3: First ranking of target health professional and patient behaviours

Total behaviour lists were presented back to participants on a screen at the front of the room. Participants privately ranked their top six health professional and top six patient behaviours on paper sheets which were collected by members of the research team.

##### Stage 4: Calculation of first group ranking, feedback and discussion

The results of the first ranking were manually entered into an excel spread sheet by members of the research team as the meeting progressed. Data entry was checked for accuracy after the meeting by a second researcher; minimal discrepancies were identified. Top ranked priority behaviours were assigned a score of 6, second ranked behaviours were assigned a score of 5 and so on. The total scores for each behaviour were calculated and the results were presented back to the group. The six most highly ranked health professional and patient behaviours in each key diabetes area were highlighted. In a group discussion, the facilitator asked participants to comment on the results, particularly focusing on behaviours whose rankings they found surprising or interesting.

##### Stage 5: Second ranking of target health professional and patient behaviours

Stage 5 followed a similar procedure as Stage 3 and participants were asked to privately re-rank top six health professional and top six patient behaviours on paper sheets in each of the three key areas.

#### Post meeting

##### Stage 6: Calculation of second group ranking

As before, top ranked priority behaviours were assigned a score of 6, second ranked behaviours were assigned a score of 5 and so on and the total scores for each behaviour were calculated. The number of times each behaviour was ranked in participants’ top six and the percentage of participants who ranked each behaviour within their top three priorities were also calculated.

##### Stage 7: Post-meeting feedback

A summary of the findings was sent to all participants three weeks after the DRP meeting. Participants were sent a link to an online questionnaire and asked to provide feedback on how interesting, enjoyable and useful they found the meeting and to give suggestions as to how the meeting could have been improved.

## Results

### Participants

Twenty-four people (10 male, 14 female) participated in the DRP process including hospital and primary care practitioners (*n* = 10), public health practitioners (*n* = 3), people with Type 1 DM (*n* = 3), people with Type 2 DM (*n* = 3), researchers in diabetes (*n* = 2), a policy leader, a patient organisation policy representative and a psychologist involved in diabetes care.

### Development of lists of health professional and patient behaviours (Stages 1–2)

Sixteen participants, including seven hospital and primary care practitioners, one public health practitioner, one diabetes researcher, one psychologist, one patient organisation representative and five patients, completed the pre-meeting online task to generate initial behaviour lists. The numbers of behaviours generated through the survey in each of the three key diabetes areas, and the numbers of behaviours following collation and additional item generation during the meeting, are shown in Table 1.

**Table 1:** Generation of health professional and patient behaviours in advance of, and during, the meeting

Key diabetes area	Total number of behaviours generated at pre-meeting survey	Number of behaviours remaining following collation	Additional behaviours generated during meeting	Final number of behaviours for ranking
Managing Type 1 DM – Patients	37	17	5	22
Managing Type 1 DM – Healthcare Professionals	42	25	7	32
Managing Type 2 DM – Patients	52	26	6	32
Managing Type 2 DM – Healthcare Professionals	47	26	5	31
Preventing Type 2 DM – General Population	46	30	8	38
Preventing Type 2 DM – Healthcare Professionals/Health Services	48	52	3	55
Total number of behaviours	272	176	34	210

### Ranking of health professional and patient behaviours in three key diabetes areas (Stages 3–6)

Table 2 shows the final highest ranked behaviours, for patients and healthcare professionals, in each of the key diabetes areas. Some participants arrived at the meeting late or had to leave early which is reflected in the different numbers of participants reported in Tables 3, 4, 5, 6 and 7. Further details on ranking results are outlined below.

**Table 2:** Highest ranked patient and health professional behaviours in three key diabetes areas

Key diabetes area	Highest ranked behaviour in Ranking 2
Managing Type 1 DM – Patients	Take insulin as required
Managing Type 1 DM – Healthcare Professionals	Engage in collaborative treatment goal setting with patients
Managing Type 2 DM – Patients	Attend and engage with structured education
Managing Type 2 DM – Healthcare Professionals	Engage in collaborative treatment goal setting with patients
Preventing Type 2 DM – General Population	Engage in healthy behaviours as a family
Preventing Type 2 DM – Healthcare Professionals/Health Services	Attend and engage with behaviour change training

**Table 3:** Ranking scores of patient behaviours within the area of managing Type 1 Diabetes Mellitus

Ranking 1 (*N* = 22)					Ranking 2 (*N* = 22)				
Rank	*Behaviours*	*Total score*	*No. of top 6 rankings*	*% of participants with item in top 3*	Rank	*Behaviours*	*Total score*	*No. of top 6 rankings*	*% of participants with item in top 3*
1	Take insulin as required	60	12	40.9	1	Take insulin as required	75	14	59.1
2	Test/monitor blood glucose as often as recommended	41	10	31.8	2	Take medication as prescribed	39	9	31.8
3	Match carbohydrates to insulin daily	37	9	31.8	3	Match carbohydrates to insulin daily	35	8	27.3
						+			
						Discussing having diabetes with others	35	8	27.3
4	Attend scheduled appointments and contacts in specialist clinic	34	9	22.7	4	Quit smoking	28	10	13.6
5	Discuss having diabetes with others	30	9	22.7	5	Attend and engage with structured education	28	9	13.6
6	Eat healthily	29	8	22.7	6	Test/monitor blood glucose as often as recommended	27	8	13.6

**Table 4:** Ranking scores of healthcare professional behaviours within the area of managing Type 1 Diabetes Mellitus

Ranking 1 (*N* = 22)					Ranking 2 (*N* = 22)				
Rank	*Behaviours*	*Total score*	*No. of top 6 rankings*	*% of participants with item in top 3*	Rank	*Behaviours*	*Total score*	*No. of top 6 rankings*	*% of participants with item in top 3*
1	Engage in collaborative treatment goal setting with patients	74	16	59.1	1	Engage in collaborative treatment goal setting with patients	72	16	54.5
2	Provide consultations that empower and motivate service users	44	10	27.3	2	Provide consultations that empower and motivate service users	57	13	40.9
3	Discuss a patient’s priorities in diabetes self-management	38	11	27.3	3	Offer structured patient education	54	13	36.4
4	Conduct annual patient screening for diabetes complications	28	9	18.2	4	Discuss a patient’s priorities in diabetes self-management	46	12	31.8
5	Provide more flexible services	28	8	18.2	5	Provide more flexible services	37	11	27.3
6	Offer structured patient education	28	8	18.2	6	Conduct annual patient screening for diabetes complications	33	9	18.2

**Table 5:** Ranking scores of patient behaviours within the area of managing Type 2 Diabetes Mellitus

Ranking 1 (*N* = 24)					Ranking 2 (*N* = 22)				
Rank	*Behaviours*	*Total score*	*No. of top 6 rankings*	*% of participants with item in top 3*	Rank	*Behaviours*	*Total score*	*No. of top 6 rankings*	*% of participants with item in top 3*
1	Attend and engage with structured education	53	15	29.2	1	Attend and engage with structured education	62	15	40.9
2	Eat healthily	49	10	37.1	2	Increase exercise	45	11	31.8
3	Engage in more self-management strategies	43	12	25	3	Engage in more self-management strategies	42	12	31.8
4	Increase exercise	32	8	16.7	4	Take medication as prescribed	37	12	18.2
						+			
						Monitor your mental health	37	9	27.3
5	Engage in physical activity, at least 30 min 5 days a week	28	6	20.8	5	Eat healthily	35	14	27.3
6	Take medication as prescribed	27	7	16.7	6	Set realistic goals for physical activity	33	8	27.3

**Table 6:** Ranking scores of healthcare professional behaviours within the area of managing Type 2 Diabetes Mellitus

Ranking 1 (*N* = 23)					Ranking 2 (*N* = 21)				
Rank	*Behaviours*	*Total score*	*No. of top 6 rankings*	*% of participants with item in top 3*	Rank	*Behaviours*	*Total score*	*No. of top 6 rankings*	*% of participants with item in top 3*
1	Conduct patient centred consultations, make sure that the patient’s needs are addressed instead of the professionals needs	48	12	30.4	1	Engage in collaborative treatment goal setting with patients	57	14	42.9
2	Engage at policy level	45	12	30.4	2	Conduct patient centred consultations, make sure that the patient’s needs are addressed instead of the professionals needs	51	12	38.1
3	Offer weight management/lifestyle modification educational programmes	44	12	30.4	3	Regularly assess patients medication, make sure patients are on optimal doses	47	14	28.6
4	Use a proactive preventative approach	43	9	30.4	4	Offer weight management/lifestyle modification educational programmes	43	11	38.1
5	Engage in collaborative treatment goal setting with patients	37	10	21.7	5	Use a proactive preventative approach	42	11	33.3
6	Conduct an annual examination of all people with Type 2 Diabetes their feet, legs and hypertension	34	10	17.4	6	Conduct an annual examination of all people with Type 2 Diabetes their feet, legs and hypertension	38	8	33.3
	+					+			
	Set more individual goals, relevant to the patient	34	8	26.1		Engage at policy level	38	10	23.8

**Table 7:** Ranking scores of population behaviours within the area of preventing Type 2 Diabetes Mellitus

Ranking 1 (*N* = 23)					Ranking 2 (*N* = 21)				
Rank	*Behaviours*	*Total score*	*No. of top 6 rankings*	*% of participants with item in top 3*	Rank	*Behaviours*	*Total score*	*No. of top 6 rankings*	*% of participants with item in top 3*
1	Engage in healthy behaviours as a family	50	12	34.8	1	Engage in healthy behaviours as a family	64	13	50.4
2	Increase exercise	29	7	21.7	2	Parental behaviours around diet and exercise for their children	45	11	38.1
3	Parental behaviours around diet and exercise for their children	28	7	21.7	3	Increase exercise	41	10	38.1
4	Reduce sedentary behaviour	26	6	17.4	4	Use smaller cups, bowls and plates to help reduce portion sizes at mealtimes	33	9	19.1
5	Use smaller cups, bowls and plates to help reduce portion sizes at mealtimes	26	7	21.7	5	Advocate for environmental change to support healthy behaviours	25	7	19.1
6	Advocate for compulsory physical education in school	26	6	13	6	Advocate for compulsory physical education in schools	24	7	23.8
						+			
						Increase cost of sugary foods	24	5	19.1

#### Managing Type 1 DM

As show in Table 3 for patient behaviours in managing Type 1 DM, ‘Take insulin as required’ was by far the highest ranked patient behaviour during both Ranking 1 and Ranking 2. Greater consensus for this behaviour was achieved during Ranking 2 when 59.1 % of participants ranked this behaviour in their top three. Interestingly, the second highest (‘Take medication as prescribed’) and fourth highest (‘Quit smoking’) behaviours in Ranking 2 did not feature in the top 6 during Ranking 1.

For healthcare professional behaviours in managing Type 1 DM, ‘Engage in collaborative treatment goal-setting with patients’ was the highest ranked behaviour during both Ranking 1 and 2, but showed slightly lower consensus at Ranking 2 (see Table 4).

#### Managing Type 2 DM

‘Attend and engage with structured education’ was the highest ranked behaviour during both Ranking 1 and 2, for patient behaviours in managing Type 2 DM. At Ranking 2, there was greater consensus and this behaviour was ranked in the top 3 by 40.9 % of participants as compared to 29.2 % of participants in Ranking 1. ‘Monitor your mental health’ and ‘Set realistic goals for physical activity’ featured in the top 6 during Ranking 2 but had not been highly ranked during Ranking 1 (see Table 5).

Healthcare professional behaviours for the management of Type 2 DM was the only category where the top ranked behaviour changed from Ranking 1 to Ranking 2. At Ranking 2, ‘Engage in collaborative treatment goal setting with patients’ was the top ranked behaviour, a jump from being ranked fifth in Ranking 1. The percentage of people ranking this behaviour in their top 3 almost doubled from 21.7 % at Ranking 1 to 42.9 % in Ranking 2. ‘Conduct patient-centred consultations, make sure that the patient's needs are addressed instead of the professionals needs’ which was ranked first during Ranking 1 was a close second in Ranking 2. The number of participants ranking this item within their top 3 also increased from Ranking 1 to Ranking2 (see Table 6).

#### Preventing Type 2 DM

‘Engage in healthy behaviours as a family’ was the top ranked population behaviour to prevent Type 2 DM in both Ranking 1 and Ranking 2. As shown in Table 7, consensus in prioritisation of this behaviour increased in Ranking 2 where it had a higher total score and was ranked in a greater percentage of participants’ top three behaviours. ‘Reduce sedentary behaviour’ was ranked fourth in Ranking 1 but did not feature in the top six in Ranking 2 and was replaced by ‘Advocate for environmental change to support healthy behaviours’ and ‘Increase cost of sugary foods’.

‘Attend and engage with behaviour change training’ was the highest ranked healthcare professional/health service behaviour at both Ranking 1 and 2. However, there was not as clear a difference between the top ranked behaviour and the second ranked behaviour ‘GPs should use weight charts to estimate BMI of children more and advise parents on the best course of action when a child is at the overweight stage’ as there was in other categories (See Table 8). The remaining behaviours in the top six were the same between Ranking 1 and Ranking 2 albeit in a slightly different order.

**Table 8:** Ranking scores of healthcare professional behaviours within the area of preventing Type 2 Diabetes Mellitus

Ranking 1 (*N* = 23)					Ranking 2 (*N* = 18)				
Rank	*Behaviours*	*Total score*	*No. of top 6 rankings*	*% of participants with item in top 3*	Rank	*Behaviours*	*Total score*	*No. of top 6 rankings*	*% of participants with item in top 3*
1	Attend and engage with behaviour change training	38	9	21.7	1	Attend and engage with behaviour change training	43	9	38.9
2	Lobby governments to fund community health programmes	37	9	26.1	2	GPs should use weight charts to estimate BMI of children more and advise parents on the best course of action when a child is at the overweight stage	40	9	44.4
3	Advocate for policy level health behaviour change	36	9	21.7	3	Advocate for policy level health behaviour change	34	8	27.8
4	Set specific targets for physical activity	22	7	17.4	4	Set specific targets for physical activity	28	6	27.8
5	GPs should use weight charts to estimate the BMI of children more and advise parents on the best course of action when a child is at the overweight stage	21	5	13	5	Provide education at schools	24	6	22.2
6	Provide education at schools	20	7	13	6	Lobby governments to fund community health programmes	20	5	16.7

### Post-meeting feedback (Stage 7)

Fifteen participants, including five hospital and primary care practitioners, two public health practitioners, one diabetes researcher, one psychologist, and four patients completed the post-meeting feedback questionnaire. Two of the respondents did not provide their details. On a scale from 1 to 5, the mean scores for how enjoyable, useful and interesting participants found the meeting were 3.9, 3.6, and 3.9 respectively (where higher scores indicate higher levels of these characteristics). Common suggestions for improvement included increasing the time for discussion, including more patients, and reducing the number of options by using broader umbrella terms to group similar behaviours together.

## Discussion

This study demonstrates that it is possible to work with a group of diverse stakeholders - people with diabetes, healthcare professionals and policy makers - in a consensus process to co-produce a prioritised list of behaviours to target for intervention research. Key stakeholders are more likely to drive forward the implementation of research findings for research topics important to them and over which they feel ownership. By engaging with key stakeholders from the outset, we have begun developing strategic alliances with health partners and service users which will be maintained throughout the programme of research.

Utilizing a nominal group process approach resulted in the identification of priorities at a broader level than could have been achieved with a more narrow focus on treatment uncertainties. Attendance and engagement with structured education, for example, would not have been identified as a priority if we had followed the guidance provided for James Lind Alliance Priority Setting Partnerships as evidence on the efficacy of structured education programmes is already available. Our approach allowed stakeholders to identify behaviours where regular performance of the behaviour, rather than treatment uncertainty, is the major problem.

The nominal group process findings indicated that in most cases, the most highly ranked behaviour was the same for Ranking 1 and Ranking 2, suggesting that the discussion and re-ranking process did not significantly change participants’ views. However, there were examples where behaviours featured in the top 6 at Ranking 2 but not at Ranking 1, presumably reflecting the impact of suggestions and arguments offered by participants during group discussions. Details of the discussions that might have led to these changes were not recorded, but would make an interesting and useful addition to future nominal group exercises. As expected in a consensus building study, consensus generally increased in relation to endorsement of the most highly ranked behaviours between the first and second rankings.

A number of striking findings emerged in relation to the most highly ranked priorities. For people with Type 1 DM there was general consensus that targeting ‘taking insulin as required’ is the most important behaviour for research. Indeed, the top three highest ranked behaviours were related to effectively monitoring and managing blood glucose, by administering insulin and counting carbohydrates. The skills required for people with Type 1 DM to maintain good glucose control are complex and demanding [21]. A number of studies report that glucose control remains unsatisfactory despite close monitoring and participation in educational programmes by many people with Type 1 DM [22], although there is some evidence that training in flexible, intensive insulin treatment can improve glycaemic control [23]. Future research should focus on developing and delivering effective interventions to promote the skills needed by people with Type 1 DM to achieve good glycaemic control.

For people with Type 2 DM, attending and engaging with structured education programmes was most highly ranked. Research into structured education programmes for people with Type 2 DM in Ireland has been lacking, but recent studies have demonstrated the efficacy of structured education in Ireland [24]. However, a significant proportion of people with Type 2 DM are either not offered or do not attend structured education programmes [25]. Future research should focus on ways to engage people with Type 2 DM in education programmes, as well as ensuring that such programmes are as patient-centred as possible to increase the benefits to participants.

Interestingly, behaviours related to collaborative goal setting and patient-centred care were highly ranked. ‘Engage in collaborative treatment goal setting with patients’ was the top ranked healthcare professional behaviour for the management of both Type 1 DM and Type 2 DM. Among people with diabetes, patients’ perceptions of collaborative care (including collaborative goal setting) have been shown to be associated with patients’ reported self-management [26], however, the relationship between collaborative goal setting and clinical control among remains poorly understood [27]. Future research should focus on interventions to promote the use of collaborative goal setting among healthcare professionals and people with diabetes, and measuring the associated impact on clinical outcomes.

In relation to preventing Type 2 DM, the most highly ranked behaviours were promotion of healthy exercise and diet, with priority given to engaging in healthy behaviours as a family. There is considerable evidence that increased levels of physical activity are related to better clinical outcomes in diabetes, and additional evidence that physical activity interventions are particularly effective when combined with dietary advice [28]. There was much discussion at the meeting around the need to advocate for policy change and attempt to promote environmental change to support healthy behaviour. There is evidence that environmental and policy approaches lead to increases in physical activity [29], and future research should focus on finding ways to maximise the role of behavioural science theory in development and implementation of public health interventions [30].

For healthcare professionals’ behaviours to prevent Type 2 DM, the highest ranked behaviour was ‘attend and engage with behaviour change training’. Recent trials of behaviour change counselling have found limited effects on behaviour change outcomes [31]. Future research should focus on finding more effective ways to train healthcare professionals to promote behaviour change among their patients.

The current study forms the first step in a behaviour change intervention development process. One behaviour relevant to people with diabetes, and one behaviour relevant to healthcare professionals, will be selected as targets for intervention development. As per recommended guidance [32], the selection of target behaviours will be informed by the comparison of the top behaviours identified in each key area under criteria of the likely impact of changing the behaviour, the likelihood of change, the potential positive or negative spillover to other behaviours and the ease of measurement of each behaviour.

### Limitations

Post-meeting feedback indicated that participants would have liked more time for discussion, although whether this would be best achieved through a longer meeting or additional meetings was unclear. Addressing priorities for both Type 1 DM and Type 2 DM on the same day may have been overly ambitious and at times the research team agreed that the meeting felt rushed. Covering Type 1 DM and Type 2 DM at the meeting reflected an attempt to reduce time and travel burden for participants who had experience of both conditions. More time for discussion might have resulted in slightly different priorities. However, when asked how they found the length of the meeting, one third of participants indicated that the meeting was too long. Finding balance between adequate time for discussion and reducing burden on participants with busy schedules is a challenge and should be considered in future research prioritisation exercises.

The nominal group technique has been recommended for groups no larger than ten people [19]. However, to ensure we addressed a range of stakeholder experiences we included different types of healthcare professionals, as well as patients and policy makers. Including this range required a larger number of participants. This may also have added to the sense of the process being rushed as group feedback took longer than with a smaller number of participants. Future nominal group technique processes that require larger numbers could consider guidance from Cantrill et al. [19] to split the sample into two or more groups and to pool results.

Consensus refers to level of agreement among participants in a given round and stability refers to level of agreement between rounds. We did not decide on a priori levels of agreement and stability required for consensus but instead chose to work through two ranking rounds within a one day meeting. The degree to which true consensus was reached is not clear, although in general the endorsement of the most highly ranked behaviours increased between the first and second rankings.

Previous discussions on the use of nominal group technique have debated the merits of mixed versus homogenous groups of participants [19, 33]. As our intention was to achieve consensus on the most important target behaviours for research in diabetes in Ireland we included a mix of patients, providers and policy makers, as each of these stakeholders have a role in behaviour change in diabetes. Despite the best efforts of the experienced facilitator, it is possible that patients may have felt less confident in voicing their opinions given the potentially hierarchical nature of provider-patient relationships. However, the initial generation of lists of behaviours and the actual ranking of behaviours were conducted privately to reduce social pressure and to allow the opinion of each participant to be given equal weighting.

Our nominal group technique process also lacks the representativeness that might be achieved with a large random sample. The overall number of participants was relatively small and, although we purposively sampled for diversity of expertise and experience, the views cannot be claimed to be nationally representative. The views of the 24 stakeholders who took part may not be typical of such a widespread condition and could not be expected represent all possible priorities. In particular, we felt it would have been beneficial to include more people with diabetes, as experiences can vary widely between individuals. Previous studies using the nominal group technique have found participants’ views to be an adequate representation of the views of the wider community [34]. However, these studies used the nominal group technique with healthcare professionals; as our process included healthcare professionals, patient and policy makers we cannot claim the 24 included participants were representative of each of these groups. Finally, the current study describes research priorities identified within the Irish health system, and caution should be used when generalising as priorities vary according to health system context [35].

## Conclusion

To impact, and improve, health and healthcare, behavioural researchers need to engage with stakeholders outside of the research community. In the current study, we have demonstrated that it is possible to engage people with diabetes, healthcare professionals and policy makers working in the area of diabetes to generate and prioritise behavioural research topics. The priorities identified were co-produced by key stakeholders, including patients, healthcare professionals and policy makers, and will inform the development of a programme of behavioural research in diabetes in Ireland. We will continue to engage with these stakeholders by inviting patients, professionals and policy makers to sit on steering committees to move chosen priorities forward. The study also provides a worked example of a research prioritisation process using the nominal group technique which may be a useful for other researchers.

## Abbreviations

DRP: Diabetes research prioritisation
Type 1 DM: Type 1 Diabetes Mellitus
Type 2 DM: Type 2 Diabetes Mellitus
